# Supplementary material for: An International Laboratory for Systems and Computational Neuroscience
Source: Neuron. 2017 Dec 20;96(6):1213–8. doi: 10.1016/j.neuron.2017.12.013 (PMC5752703; doi:10.1016/j.neuron.2017.12.013)
Supplement: Document S1. Author Affiliations [file mmc1.pdf]

Neuron, Volume *96*

Supplemental Information

An International Laboratory for Systems and Computational Neuroscience

The International Brain Laboratory

Author affiliations:

Larry F. Abbott, Columbia University
Dora E. Angelaki, Baylor College of Medicine
Matteo Carandini, University College, London
Anne K. Churchland, Cold Spring Harbor Laboratory
Yang Dan, Howard Hughes Medical Institute, University of California, Berkeley
Peter Dayan, University College, London
Sophie Deneve, École Normal Supérieure
Ila Fiete, University of Texas at Austin
Surya Ganguli, Stanford University
Kenneth D. Harris, University College, London
Michael Hausser , University College, London
Sonja Hofer, Sainsbury Wellcome Centre for Neural Circuits and Behaviour, Univ. College London
Peter E. Latham, University College, London
Zachary F. Mainen, Champalimaud Research
Thomas Mrsic-Flogel , Sainsbury Wellcome Centre for Neural Circuits and Behaviour, Univ. College London
Liam Paninski, Center for Theoretical Neuroscience, Columbia University
Jonathan W. Pillow, Princeton University
Alexandre Pouget, University of Geneva
Karel Svoboda, Janelia Research Campus
Ilana B. Witten, Princeton University
Anthony M. Zador, Cold Spring Harbor Laboratory
