# Supplementary material for: An International Laboratory for Systems and Computational Neuroscience
Source: Neuron. 2017 Dec 20;96(6):1213–8. doi: 10.1016/j.neuron.2017.12.013 (PMC5752703; doi:10.1016/j.neuron.2017.12.013)
Supplement: Document S2. Article plus Supplemental Information [file mmc2.pdf]

CellPress

An International Laboratory for Systems and Computational Neuroscience

The International Brain Laboratory*
*Correspondence: churchland@cshl.edu
https://doi.org/10.1016/j.neuron.2017.12.013

The neural basis of decision-making has been elusive and involves the coordinated activity of multiple brain structures. This NeuroView, by the International Brain Laboratory (IBL), discusses their efforts to develop a standardized mouse decision-making behavior, to make coordinated measurements of neural activity across the mouse brain, and to use theory and analyses to uncover the neural computations that support decision-making.

Introduction

Making a decision requires processing sensory information, evaluating and predicting rewards, integrating past experience, selecting actions, and executing them. The neural basis of these processes has been elusive, likely because they are mediated by multiple brain structures working together. The relevant signals are thus distributed over large neuronal populations spread across the brain. To overcome these challenges, the International Brain Laboratory (IBL) seeks to standardize and reproduce one decision-making task in the mouse, and make multiple neural measurements to achieve dense coverage of the mouse brain at the neuronal level.

We are a collection of experimentalists and theorists who are recruiting a team of talented trainees working closely together across the boundaries of individual laboratories. Experimental laboratories will standardize a steering-wheel task for head-fixed mice, to probe decisions based on visual perception and on history of reward. They will then record from many different brain areas using multiple recording modalities to build up a dense dataset of activity measurements during the task. Theoretical laboratories will harness this unprecedented dataset, contributing computational expertise and developing and testing new theories based on multi-region interactions.

This approach differs from traditional neuroscience, in which individual labs work with different behaviors and record from a small number of brain areas. In other areas of science, such as physics and genomics, large teams have been successfully collaborating on large-scale projects for years. Bringing this collaborative approach to neuroscience will pose important challenges, but it will also allow our field to likewise harvest the benefits of working collectively on difficult scientific problems.

Understanding the Brain Demands a Collaborative Approach

Behavior is generated by patterns of activity in large groups of neurons distributed across brain regions. Understanding the structure of these representations, how they are learned, and how they lead to behavior has enormous potential benefits.

This frontier is now within reach, thanks to powerful new tools that are being harnessed by a growing neuroscience community. The community of neuroscientists is substantial: the annual meeting of the United States Society for Neuroscience draws over 25,000 scientists, whose expertise covers a plethora of brain functions, brain regions, and techniques. Recent innovations have provided this community with tools to record and analyze the activity of neuronal populations with unprecedented specificity and scale (Sofroniew et al., 2016; Jun et al., 2017; Paninski and Cunningham, 2017).

However, the power of these resources and tools is not yet fully utilized because individual laboratories typically pursue problems in relative isolation (Mainen et al., 2016). They apply the new tools piecemeal, to study the activity of neurons in one or two brain regions at a time. Moreover, they typically develop their own bespoke versions of methods and approaches—such as unique behavioral paradigms, definitions of brain regions, and customized recording and analysis methods—making it difficult to compare, reproduce, and synthesize results across laboratories. Even when two laboratories study nominally the same task, the attempt to compare their results can result in controversy, with small differences in methods obscuring the possibility of correspondences. Indeed, experiments in systems neuroscience are rarely replicated.

Overall, there is a disparity in scale between the brain's complexity and the efforts of individual neuroscience laboratories. Lone laboratories lack the resources and capacity to study the large set of regions, connections, and cell types involved in even one behavior. While the work of individual laboratories remains essential for exploratory study, the piecemeal approach seems insufficient to make full use of the new tools that are available, to produce an overall understanding of brain function.

A Collaboration of 21 Laboratories with Joint Trainees

To surmount these obstacles, the IBL pools the expertise of 21 experimental and theoretical laboratories aiming to reveal the processes that support decision-making. We seek to understand these processes at the neuronal level, and at both micro- and macro-scales, so that we can elucidate the role of local circuits and brain regions, and the dynamic interactions between regions. The team's experimentalists will collect data from numerous brain areas and will pool these

data to obtain dense coverage spanning the entire brain at the neuronal scale. Experimentalists and theorists will work closely together to analyze the data and build new theories about the underlying brain-wide circuits.

To recruit the best postdocs to the project, we have established a framework to allow IBL postdocs to build an internationally competitive research CV while also reaping the unique benefits of working on a large-scale collaborative project. In the project's early months, while the behavioral task and recording methods are being established in each lab, postdocs will contribute data and/or analyses to a large-scale brain activity map covering the entire brain at low resolution. After this initial stage, each postdoc can lead his or her own hypothesis-driven project focusing in depth on a specific question—for example, the role of a particular brain region in the common behavioral task. These hypothesis-driven projects will be chosen by the postdocs according to their own interests and will benefit from the shared resources (hardware, software, analysis tools and ideas, training expertise, reagents, etc.) that emerge from the IBL collaboration. Collaborations between IBL postdocs (for example, theorists and experimentalists) will be encouraged and are expected to arise organically, catalyzed by regular in-person and video project meetings. Norms for credit assignment on such collaborations will be similar to those that currently operate within a single lab. Each postdoc is therefore expected to be first author on papers resulting from his or her own hypothesis-driven project, as well as contributing author on a major paper describing the large-scale activity map and multiple hypothesis-driven papers led by other postdocs.

In addition, IBL will provide a support network to ensure that postdocs and other scientists obtain the credit and visibility they need to advance their careers. Support will include guidance and advocacy by established faculty members, who can communicate the specific contributions of an IBL scientist to a search committee. This will provide an advantage for IBL postdocs compared to postdocs from traditional labs, who have only a single mentor to advocate for their postdoctoral achievements. This kind of approach is successful in genomics: papers involving the entire ENCODE consortium have as many as 300 authors (ENCODE Project Consortium et al., 2007; Djebali et al., 2012) and the collaboration is seen as an attractive option for postdocs.

Probing the Neural Basis of a Decision-Making Behavior

The IBL aims to understand the neural basis of decision-making in a behavioral task performed by head-fixed mice. We chose mice because they allow the study of mammalian brain and behavior with ease of experimental control and accessibility, and access to a growing arsenal of atlases, databases, and genetic tools. Mice, moreover, have emerged as a leading species for studying decision-making because they exhibit stable and reliable behavior (Carandini and Churchland, 2013). Head-fixing, in turn, provides ready access to the brain and stable conditions for recording and imaging, and allows continuous control of visual inputs and knowledge of eye position.

We will initially focus on a single basic task, designed to probe decisions based on visual perception and on history of reward (Figure 1). In the task, head-fixed mice turn a steering wheel to indicate whether a visual stimulus appears to their left or to their right to obtain a water reward (Figure 1B) (Burgess et al., 2017). In different trials, the stimulus is made easier or harder to detect, e.g., by changing its contrast. This way, the mouse will make mistakes and will occasionally have to guess (Figure 1C). These mistakes and guesses are highly informative for investigating the neural basis of perceptual decisions. On a slower timescale (e.g., across blocks of trials), one of the two choices (left or right) is made more valuable than the other by changing the relative reward. To maximize reward, the mouse thus has to modify its choices to stimuli that are perceptually uncertain, but not to stimuli that are more certain (Figure 1C). The reward environment is thus dynamic, encouraging animals to base decisions not only on sensory properties of the stimulus, but also on their internal knowledge of the reward structure.

Our first aim is to standardize this basic task and replicate it across laboratories, ideally obtaining indistinguishable behavioral performance across laboratories. This will ensure that we can obtain neural recordings in fully comparable behavioral conditions and thus pool the resulting data. From this basic task, at later stages of the project we will also derive branches: task innovations that will be essential for testing new hypotheses for the postdoc-led projects. Hardware and software specifications of the task will be openly available, so that laboratories outside IBL will also be able to use and extend the task for their own studies.

A Multi-modal Approach to Neural Measurements

To understand how brain-wide neural activity supports these decisions, we will record activity during behavior using multiple modalities. We will aim for comprehensive coverage of the brain, sampling activity at the neuronal level. Individual labs will record in agreed locations, each one duplicated in a separate lab so that results can be compared and replication assessed, before the data from all laboratories are pooled into a single database.

We will use three complementary recording techniques (Figure 2). First, we will use Neuropixels probes to simultaneously measure single-neuron spiking activity with millisecond precision across many brain areas (Jun et al., 2017; Figure 2A). The 1,000 sites of these probes are arranged over 1 cm, allowing one to record large numbers of neurons from different brain areas simultaneously. Pooling these measurements across labs will then generate a brain-wide picture of neural activity. For example, at a 0.5 mm grid spacing, IBL experimentalists can make recordings that together cover the brain in <100 penetrations, each spanning the brain's full depth. The combined high temporal resolution and broad spatial coverage of this technique will reveal how and where decision-related signals change over time and interact across regions as an animal commits to a choice.

Second, we will make cortex-wide recordings of neurons tagged with genetically encoded Ca^{2+} indicators by imaging with recently developed two-photon mesoscopes (Sofroniew et al., 2016; Figure 2B). This technique will allow us to track populations of neurons longitudinally, as animals go from being novice to expert decision-makers.

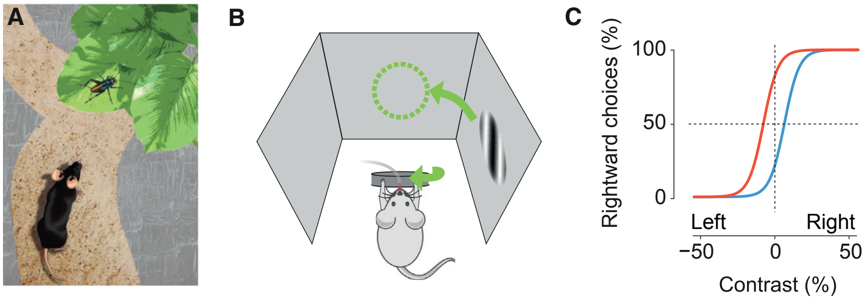

Figure 1. Probing Decisions Based on Perception and Value in Head-Fixed Mice
(A) Finding prey requires making decisions based on sensory systems and on prior experience of cost and value.
(B) A laboratory task to probe decision-making. Mice select a visual stimulus and report their choice by moving the stimulus to the center with a steering wheel (Burgess et al., 2017).
(C) Schematic of mouse decisions as a function of stimulus strength (e.g., visual contrast). When the stimulus is strong (50% contrast left or right), it drives most choices. When the stimulus is weak or absent (0 contrast), choices depend on whether rewards are larger for rightward choices (red) or leftward choices (blue).

Third, we will measure neuromodulatory activity using photometry and microendoscopy (Figure 2C). The use of these approaches to record from genetically encoded Ca^{2+} indicators will result in cell-type-specific and relatively high temporal resolution signals from deep brain structures. In particular, this will make it possible to record from four major neuromodulatory systems: serotonin, dopamine, norepinephrine, and acetylcholine. These data will show how neuromodulators support decision-making on the relevant timescales, while revealing similarities and differences across neuromodulatory populations.

All of these experimental approaches are already being used in many of our laboratories. The novel aspect of the IBL is that they will be combined in a unified way, assuring complementary views of brain-wide neural activity that can be harnessed systematically to produce new insights. Data from all recording modalities will be stored in a single database, where it can be pooled and analyzed together. Furthermore, it will be made globally accessible once the results are published.

In a subsequent stage of the project, these measurements will then be used to identify areas of interest for future in-depth studies. The data will inspire hypothesis-based experiments, which will target particular brain circuits. These could include causal manipulations (e.g., optogenetic silencing and activation) and more precise cellular recordings (e.g., whole-cell *in vivo* patch clamp).

Developing Hypotheses and Analytical Frameworks

Theoretical and experimental neuroscientists will collaborate on all aspects of IBL research, from the conception, design, and execution of experiments through to their analysis and interpretation. The adoption of a single task and the coordinated recording of activity in many areas simultaneously provide two particular opportunities for IBL theorists.

The first is to take advantage of the substantial body of data on every choice of many individual animals to build a more complete account of a single moderately complex behavior involving the processing of uncertainty and reward. This should elucidate differences in strategies between subjects and path dependencies in learning, along with an understanding of the goals different subjects pursue. A quantitative understanding will lay the groundwork for the subsequent construction of circuit models of the neural dynamics that underlie the behavior.

The second opportunity is to exploit the resulting multiple recordings to examine coordinated activity across many regions and areas—i.e., the dynamics of how information and decision-related signals could be processed, gated, and transmitted within and between areas to achieve the behavioral benchmarks measured earlier. Furthermore, the quantification of the trajectories of behavioral competence will be married to that of the neural changes that are responsible.

As is conventional, theorists will take as input empirical results, while contributing hypotheses for experiments to be performed within IBL, providing a unique opportunity for answering previously severely under-constrained questions about how multiple areas and signals cooperate in a time-resolved way to perform a unified computation. Throughout the project, theorists will develop data analytic tools and help construct the data visualization pipeline, to ensure tight coordination between theory and experiment (Paninski and Cunningham, 2017). To further facilitate theory-experiment and theory-theory collaborations, multiple theory group members will be embedded in experimental laboratories for coordinated scientific visits.

Architecture for Continuous, Long-Term Data Access

Data sharing is critical to the goals and success of the IBL program. To this end, we will adopt the best practices available for data storage, sharing, and analysis. Specifically, we aim to optimize two features of data architecture and make the results open source.

First, we are developing a pipeline for data sharing, visualization, and storage (Figure 3). The pipeline starts with raw neural data that are preprocessed and compressed after each experiment. The data and metadata are then placed in a standardized format adopted by all laboratories so that we can collate large raw data files from multiple laboratories and implement the same analysis routines regardless of the data's origin (Teeters et al., 2015). Once in this format, data will be uploaded to a database structured so that users can identify and download relevant datasets with ease. This database structure will allow us to easily share the data globally, following the lead of the Allen Institute (http://observatory.brain-map.org/visualcoding/).

Second, we are developing shared data analysis tools. These include signal processing pipelines that detect neuronal activity from raw images of calcium fluorescence or raw electrode signals (Figure 3, left). Once neural activity traces (along with quality measures) are extracted from the raw data, we will apply existing and newly developed dimensionality reduction and Bayesian analysis methods

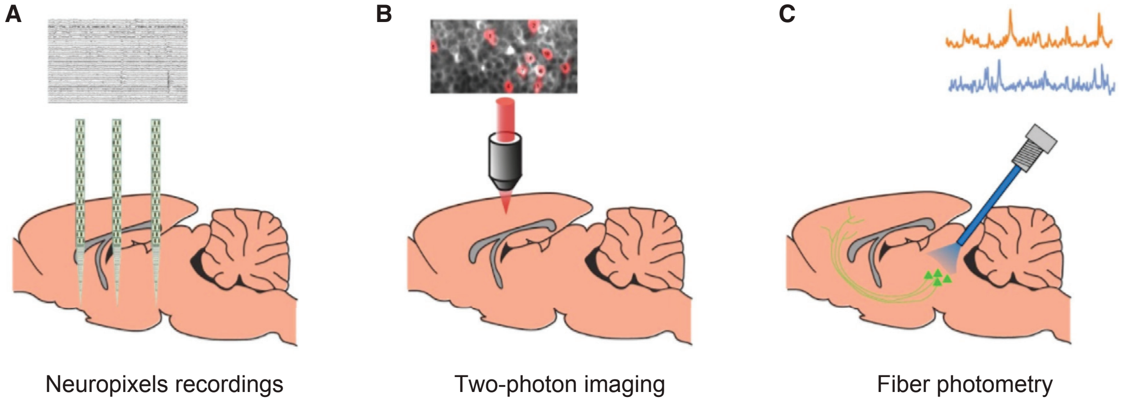

Figure 2. Three Modalities for Measuring Neural Activity
(A) Neuropixels recordings from multiple brain areas.
(B) Two-photon imaging across cortical regions.
(C) Fiber photometry of neuromodulator pathways.

to link the high-dimensional neural recordings with interpretable theoretical models of multi-region neural dynamics (Figure 3, right). Again, these tools will be made publicly available.

Ambitious, Open-Ended Goals Require Large-Scale Collaboration

Large-scale collaborations have successfully addressed difficult problems in multiple domains of science. These range from high-energy particle physics, exemplified by CERN, in which hundreds of laboratories are brought together through the use of a piece of very large-scale experimental hardware, to biology, exemplified by the Human Genome Project (HGP) or, more recently, the Human Connectome Project (HCP), both of which had goals that could be broken into individual lab-sized pieces, and for which a joint solution could be achieved by assigning particular pieces to particular labs.

Within neuroscience, collaborations have historically been smaller and rarer than in physics and genomics. This is perhaps because in systems neuroscience it is harder to define ambitious goals that can benefit from a tight collaboration and have clear end points. Within IBL, for example, the goal of understanding how neural systems support a complex behavior will naturally lead to new hypotheses; a single end point cannot be fully specified from the outset.

Despite these challenges, collaborative efforts in neuroscience are emerging. Examples within academia include the BRAIN initiative, the IARPA MICRONS project, the HCP, the NSF NeuroNex projects, and the Human Brain Project (Yuste and Bargmann, 2017). These efforts bring together larger teams of researchers to develop new technologies and drive forward our understanding of brain function. IBL is much smaller and it has a singular focus: mapping brain-wide activity at single-neuron resolution during a single behavioral task.

Outside academia, organizations like the Allen Institute for Brain Sciences and HHMI's Janelia Research Campus are dedicated to accelerating neuroscience and are leaders in standardization of procedures and in data sharing. However, these too differ from IBL in key ways. For instance, IBL is distributed geographically, a network of laboratories across the world that represent complementary expertise for the scientific goal at hand. To best coordinate these laboratories, IBL adopted a governance that differs from the typical top-down structure. Rather, our governance is inspired by the ATLAS collaboration at CERN, which encourages a participatory, collaborative, and non-hierarchical decision-making process.

Although IBL differs from these neuroscience collaborations, we seek opportunities to integrate our efforts with theirs. Combining efforts will allow teams to more effectively tackle common challenges, such as data sharing, cloud storage, cloud computing, and project management. Further, adoption of a common data format across collaborations will greatly extend the reach of each, and will encourage standardization in the field at large.

Challenges

Many aspects of our project constitute challenges that we will have to overcome. A first set of challenges concerns the customs of research laboratories in neuroscience. The efforts we devote to our individual laboratories and to IBL need to be balanced, and may well give rise to occasional conflict requiring resolution. Simply maintaining a true collaboration between 21 laboratories accustomed to going their own way will be a major novelty in neuroscience.

A second set of challenges arises from the sheer difficulty of replicating the same exact mouse behavior across ten experimental laboratories, achieving such a uniformity in results that behavioral data from different laboratories will be indistinguishable. This is an ambitious goal, and achieving it will itself be an important milestone, before even the first spike is recorded.

There are, of course, many other challenges, but another one that deserves mention is the importance of maintaining openness to the rest of the field, so that our collaboration is not seen as a competitor but rather as a positive source of open standards, methods, data, and ideas.

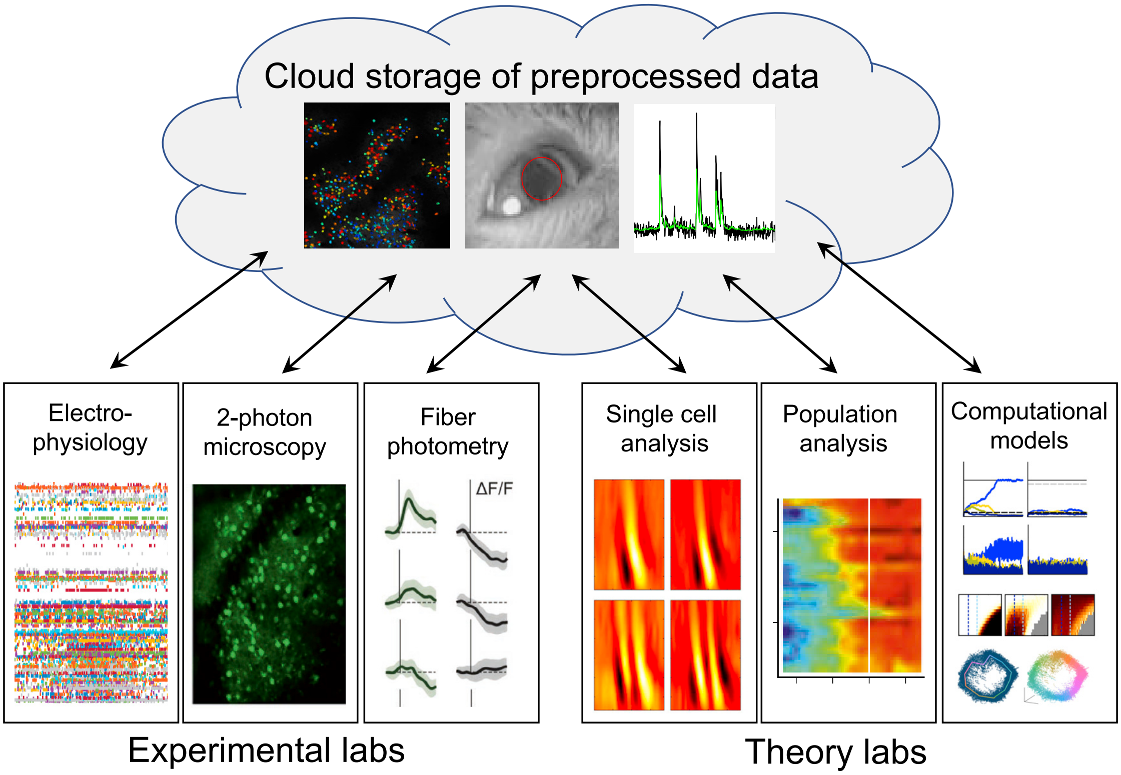

Figure 3. Architecture for Sharing Data Collaboration-Wide Using a Cloud-Based System
Experimentalists collect data in three modalities (left), which is then preprocessed and uploaded to a cloud-based server. All members of the collaboration then have full and immediate access to the data for analysis and modeling (right).

Perspectives

The International Brain Laboratory joins together diverse experimental and theoretical neuroscience teams to pursue a common goal: to develop a unified brain-wide theory of a complex behavior, at the neuronal level. Though this goal lies in the domain of fundamental research, achieving it could have major scientific and societal impact. Indeed, the results are likely to be relevant for understanding psychiatric diseases and for driving further research in robotics and artificial intelligence. We also hope that the creation of a network with a common scientific goal will foster international cooperation in neuroscience and catalyze alliances among recently launched international brain projects.

In addition to its scientific objectives, our collaboration aims to change the scientific culture in neuroscience, inspired by what has already been achieved in other fields. We wish to develop a new way of doing neuroscience in partnership across multiple laboratories, sharing experimental protocols, data, and analyses to ensure tight collaboration and high reproducibility. The open framework we are establishing should allow any laboratory to adopt or access our behavioral task, data infrastructure, and a body of data to guide and test their own hypotheses. In this way, IBL can become both a template and a platform for collaboration, accessible by and inspiring the wider neuroscience community.

SUPPLEMENTAL INFORMATION

Supplemental Information includes a complete author list with affiliations and can be found with this article online at https://doi.org/10.1016/j.neuron.2017.12.013.

CONSORTIA

The members of the International Brain Laboratory are Larry F. Abbott, Dora E. Angelaki, Matteo Carandini, Anne K. Churchland, Yang Dan, Peter Dayan, Sophie Deneve, Ila Fiete, Surya Ganguli, Kenneth D. Harris, Michael Häusser, Sonja Hofer, Peter E. Latham, Zachary F. Mainen, Thomas Mrsic-Flogel, Liam Paninski, Jonathan W. Pillow, Alexandre Pouget, Karel Svoboda, Ilana B. Witten, and Anthony M. Zador.

ACKNOWLEDGMENTS

The International Brain Laboratory is funded by the Wellcome Trust, the Simons Foundation, and the INCF. We thank David Tank and Carlos Brody for advice during the formation of our collaboration.

REFERENCES

Burgess, C.P., Lak, A., Steinmetz, N.A., Zatka-Haas, P., Bai Reddy, C., Jacobs, E.A.K., Linden, J.F., Paton, J.J., Ranson, A., Schröder, S., et al. (2017). High-yield methods for accurate two-alternative visual psychophysics in head-fixed mice. Cell Rep. *20*, 2513–2524.

Carandini, M., and Churchland, A.K. (2013). Probing perceptual decisions in rodents. Nat. Neurosci. *16*, 824–831.

Djebali, S., Davis, C.A., Merkel, A., Dobin, A., Lassmann, T., Mortazavi, A., Tanzer, A., Lagarde, J., Lin, W., Schlesinger, F., et al. (2012). Landscape of transcription in human cells. Nature *489*, 101–108.

ENCODE Project Consortium, Birney, E., Stamatoyannopoulos, J.A., Dutta, A., Guigó, R., Gingeras, T.R., Margulies, E.H., Weng, Z., Snyder,

M., Dermitzakis, E.T., Thurman, R.E., et al.; NISC Comparative Sequencing Program; Baylor College of Medicine Human Genome Sequencing Center; Washington University Genome Sequencing Center; Broad Institute; Children's Hospital Oakland Research Institute (2007). Identification and analysis of functional elements in 1% of the human genome by the ENCODE pilot project. Nature *447*, 799–816.

Jun, J.J., Steinmetz, N.A., Siegle, J.H., Denman, D.J., Bauza, M., Barbarits, B., Lee, A.K., Anastassiou, C.A., Andrei, A., Aydın, Ç., et al. (2017). Fully integrated silicon probes for high-density recording of neural activity. Nature *551*, 232–236.

Mainen, Z.F., Häusser, M., and Pouget, A. (2016). A better way to crack the brain. Nature *539*, 159–161.

Paninski, L., and Cunningham, J. (2017). Neural data science: accelerating the experiment-analysis-theory cycle in large-scale neuroscience. bioRxiv. https://doi.org/10.1101/196949.

Sofroniew, N.J., Flickinger, D., King, J., and Svoboda, K. (2016). A large field of view two-photon mesoscope with subcellular resolution for in vivo imaging. eLife *5*, https://doi.org/10.7554/eLife.14472.

Teeters, J.L., Godfrey, K., Young, R., Dang, C., Friedsam, C., Wark, B., Asari, H., Peron, S., Li, N., Peyrache, A., et al. (2015). Neurodata without borders: creating a common data format for neurophysiology. Neuron *88*, 629–634.

Yuste, R., and Bargmann, C. (2017). Toward a global BRAIN initiative. Cell *168*, 956–959.

Neuron, Volume *96*

Supplemental Information

An International Laboratory for Systems and Computational Neuroscience

The International Brain Laboratory

Author affiliations:

Larry F. Abbott, Columbia University
Dora E. Angelaki, Baylor College of Medicine
Matteo Carandini, University College, London
Anne K. Churchland, Cold Spring Harbor Laboratory
Yang Dan, Howard Hughes Medical Institute, University of California, Berkeley
Peter Dayan, University College, London
Sophie Deneve, École Normal Supérieure
Ila Fiete, University of Texas at Austin
Surya Ganguli, Stanford University
Kenneth D. Harris, University College, London
Michael Hausser , University College, London
Sonja Hofer, Sainsbury Wellcome Centre for Neural Circuits and Behaviour, Univ. College London
Peter E. Latham, University College, London
Zachary F. Mainen, Champalimaud Research
Thomas Mrsic-Flogel , Sainsbury Wellcome Centre for Neural Circuits and Behaviour, Univ. College London
Liam Paninski, Center for Theoretical Neuroscience, Columbia University
Jonathan W. Pillow, Princeton University
Alexandre Pouget, University of Geneva
Karel Svoboda, Janelia Research Campus
Ilana B. Witten, Princeton University
Anthony M. Zador, Cold Spring Harbor Laboratory
